# Animal and their products used for treatment and prevention of disease practiced by traditional healers in Jimma Arjo district, East Wollega Zone, Western Ethiopia

**DOI:** 10.1002/vms3.1277

**Published:** 2023-09-19

**Authors:** Debela Abdeta Efa

**Affiliations:** ^1^ College of Veterinary Medicine and Agriculture Addis Ababa University Bishoftu Ethiopia

**Keywords:** disease, ethnozoology, fidelity level, knowledge, medicinal animals, use value

## Abstract

**Background:**

Ethiopia is one of the countries with richest fauna used for medicinal purpose. The Jimma Arjo community has knowledge and practice of utilizing this medicinal animal for treatment of human and animal ailments.

**Objective:**

The objective of this research is to assess animals and their products used for treatment of human and animal ailment

**Methods:**

A cross‐sectional ethnozoological survey was conducted using a semi structured questionnaire among purposively selected traditional healers resided in Jimma Arjo district. The data collected was entered in Microsoft excel spread sheet and analysed using SPSS statistical software. Fidelity level (FL), use value and informant consensus factor was determined.

**Results:**

A total of 33 animal species was found to be used for treating 40 human ailments and different livestock disease confirmed by 36 informants of different ages, sexes and educational backgrounds. The majority of animals (63.63%) were mammals followed by birds (15.15%). Most of the respondents were male, married and aged 55 years and above. Most of the healers learn ethnozoological practice from the father (36.11%) followed by mother (19.44%). The traditional practice is accepted by (72.22%) of the community where 61.11% of the healers are not willing to transfer their knowledge to other. For most of the healers, the reason to practice traditional healing was for treating one's own family or animals (41.67%). The FL was found 100% for honey from *Apis mellifera* and *Trigona* spp. for asthma, *Cynopterus sphinx* for viral skin disease and *Crocuta crocuta* for bad sprit treatment. In this study, honey from *A. mellifera*, *Hystrix cristata* and *Sus scrofa domesticus* were reported to cure different ailments.

**Conclusion:**

This study indicated wide use of medicinal animals and their products which could be used as an alternative and complementary medicine or a basis for in developing new drugs because the existing drugs especially antimicrobials are under threat due to the development of resistance by microbes.

## Graphical abstract

This finding assessed animals and their products used for the treatment of human and animal ailments. Collected from 36 key informants indicated a total of 33 animal species was used for treating human and livestock disease. Different healer's anonymously agreed on medicinal use of *Apis mellifera*, *Hystrix cristata* and *Sus scrofa domesticus*.

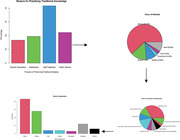

## 1 INTRODUCTION

People with different cultures across the globe apply their indigenous knowledge to prevent and treat various diseases of human and animals by using plant and animal derived remedies (Borah & Prasad, 2017). For the majority of the world's population, traditional medicinal remedies and medical practices are a primary source of health care, making indigenous medicinal knowledge an essential part of the health care system (World Health Organization, 1993). Various animals, their parts and products of both wild and domestic animals are used to prepare curative, protective and preventive remedies for a variety of ailments (Yirga et al., 2011). Animals and the products derived from their body organs constitute part of the inventory of medicinal substances which are used widely by the people since time immemorial, and such practices still exist in traditional medicines (Alves & Rosa, 2005).

Zootherapy is a form of healing that uses medicine obtained from animals to treat human or animal ailments (Costa‐Neto, 2005). All over the world, it is used in healing practices, magic rituals and religions (Anageletti et al., 1992; Rosner, 1992). As one of many known therapies practiced worldwide, zoo therapy is a viable alternative in modern society (Alves & Rosa, 2005). Marques (1994) stated that all human culture which presents a structured medical system utilizes animals as medicines. The phenomenon of zootherapy is marked both by a broad geographical distribution and very deep historical origins. Of the 252 essential chemicals that have been selected by the World Health Organization, 8.7% come from animals (Alves & Rosa, 2005). Traditional healing methods involving hundreds of insect and other invertebrate species are reviewed by Meyer‐Rochow (2017). In traditional Chinese medicine, more than 1500 animal species had been recorded to be having some medicinal use (CNCTHM, 1995). In Brazil, Alves and Rosa (2007) reported the medicinal use of 283 animal species for the treatment of various ailments. In Bahia state, in the northeast of Brazil, over 180 medicinal animals have been recorded (Unnikrishnan, 1998).

Africa has the richest and most diversified fauna resources. The continent is known to be the home of approximately 2000 important biodiversity sites and the worlds most diversified and numerous big ungulate (hoofed mammal) populations (Ripple et al., 2015; Wolf & Ripple, 2016) and freshwater fishes than any other continent. For example in South Africa, animals and plants are commonly used as traditional medicines for both the healing of ailments and for symbolic purposes such as improving relationships and attaining good fortune (Whiting et al., 2010).

Ethiopia is known for having wide climatic and ecologic conditions which possess a wide range of fauna and flora of different species that are used for medicinal purpose (Yirga et al., 2011). According to Bekele and Yalden (2013), there are more than 860 species of birds, 320 species of mammals, 200 reptiles, 145 fishes in Ethiopia and 63 amphibians. Approximately more than half of the human population in this country depends on traditional medicine for meeting their primary health care needs and 90% of livestock disease (Birhanu et al., 2015). Enormous work has been done on ethno botany and traditional medicine in the country but reports about ethno zoology of Ethiopia are still limited. From animal source molecules like heparin, insulin and barbiturate, pituitary hormone was some of the substances employed for pharmacotherapeutic effects. Insulin was first isolated from pancreases of pigs or cows, used to treat diabetic in patients in 1920. The initial source of heparin was dog liver which is used till today for variety of coronary and cardiovascular disease. Salmon sperm was used to create protamine sulphate made from utilizing recombinant DNA technology. Captopril, the first angiotensin converting enzyme inhibitor, was discovered 1970, derived from Brazilian snake *Bothrops jararaca* venom. Eptifibatide is a glycoprotein; potent inhibitor of receptor IIb/IIIa used to avoid thrombosis was extracted from rattle snake venom called *Sistrurus miliarius barbouri*. Armour thyroid is one of the most well‐known medications derived from pig thyroid glands used for the treatment of hypothyroidism. Gelatin, a substance utilized in nearly every home, is frequently used in the pharmaceutical sector to make capsules. It can be either of porcine or bovine origin (https://recipe‐cpsa.com/en/medicines‐of‐animal‐origin/). Loss of traditional knowledge of indigenous communities had impact the development of modern medicine. It is important to document the traditional knowledge of human communities, as the majority of such communities are losing their socioeconomic and cultural characteristics. The present study was carried out to investigate the use of traditional medicine of animal origin used for healing human and livestock ailments that has been practiced by people in Jimma Arjo community.

### 1.1 Gap and Justification

The Didessa basin of Jimma Arjo district is known by hot climatic condition with poor road and infrastructure like veterinary and human clinics. In addition to the original residents of the area, there are settlers from North Showa, Western Hararge and Wollo owning different religions who had long experience of using traditional medicine. Despite huge experience of the society on the use of traditional medicine, there is no documented research on use of animals as a traditional medicine for human and animal ailments from this area. As traditional knowledge is seen as a major component of Ethiopian social legacy where it is under researched and in danger of being lost. Recognizing that the natural assets from wild and domestic animals are better therapeutic options and hence to be recorded. Hence, the objective of this research is to assess traditional healing practice exercised by Jimma Arjo districts community used to cure human and livestock ailment through interviews with purposively selected respondents.

## 2 MATERIALS AND METHODS

### 2.1 Study area

The study was conducted in Jimma Arjo district of East Wollega administrative zone of Oromia regional state western part of Ethiopia bordering to Didessa river valley basin. It is bordered by Dabo Hana district in west, Bedele district in south, Nunu Kumba in north and Leka Dulecha district in east. The district is found 379 km away from Addis Ababa the capital of Ethiopia. The annual mean temperature for most part of the district is 15.7–24°C, and the elevation varies from 1200 to 2500 m above sea level (mas1) with mean annual rain fall of 824–2616 mm. The livestock populations that are found in Jimma Arjo district include cattle 112,101, sheep 30,546, goat 20,821, horses 1115, mule 315, donkey 10,424 and poultry 63,902. The district has human population of around 156,800. Among these animals cattle are the dominant species raised in the area.

The vegetation type of the area is characterized by common riverine vegetation. The area has been a virgin and with reserved vegetation. It is rich with wild game animals in many river systems and savanna. Some of these wild animals include baboons, monkey, African buffalo, lion, bush pig, crocodile, hyena, snakes and others (Livestock development and Fisher resource Office of the district, 2019).

The farming system of the district is mixed farming system, from total population of the district 89% engage in agriculture and livestock production. The common crop of the area are coffee, teff, sorghum, maize, wheat, barley, and inset, sesame, ginger and rice are also planted in some extent. Honey production and chat plantation are also commonly practicing. Natural broad leaf forests and grasslands cover non‐cultivated lands in the area. The main farming system in the area is mixed farming, and most abundant animal species kept are cattle.

### 2.2 Methodology

Study design: In order to acquire ethnozoological information about the medicinal animal and their products used in traditional medicine, a cross‐sectional study was conducted from September 2020 to October 2021.

Sampling technique: The ethnozoological data (local name of animals, mode of preparation and administration, part of the animal used and ingredients added) were collected through semi‐structured interview prepared in English and then translated to Afaan Oromo for suitability after a pilot study was conducted for reliability of the designed questionnaire. The selection of informant's was based on their experience, recognition as experts and knowledge concerning traditional medicine and their age ranged from 25 years and above.

The healers were selected from each kebeles of the districts based on their prior experience. They were inquired, about the illnesses cured by animal based medicines and the manner in which the medicines were prepared and administered. They were also requested thorough information about mode of preparation and blending of animal products used as ingredients and whether they use animal in the healing practice, as this type of information indicate how a given medicine can be therapeutically effective in term of the right ingredients, the proper dose and the right length of medication. During the course of the study, each participant was visited at least twice in order to confirm the reliability of the ethnozoological information. Consequently, the response of an informant that was not in accord with each other was considered unreliable and hence rejected.

### 2.3 Data analysis

The data obtained was summarized and analysed using descriptive statistical methods, presented in tables as percentages. R‐studio was used for analysis purpose. In the ethnozoological data that was obtained from the interviews on reported medicinal animals and associated knowledge, fidelity level (FL) was calculated as the percentage of respondents claiming the use of a certain animal species for the same ailments, for the most frequently reported diseases or ailments as FL% = NP × 100/*N*; where NP is the number of respondents that claim use of a species to treat a particular disease, and *N* is the number of respondents that use the animals as a medicine to treat any given disease (Alexiades, 1996). The range of FL is from 1% to 100%; high values indicate that this particular animal species is used by large number of the people, whereas a low value shows that respondents disagree on the usefulness of a species in treating ailments. Moreover informant consensus factor (ICF) was calculated using the following formula, ICF = nur − nt/nur − 1, where ICF is the informant consensus factor, nur is the number of used citations in each category, and nt is the number of species used.

## 3 RESULTS

### 3.1 Socio demographic characteristics of respondents

The present study identified the traditional medicinal knowledge of treating various diseases using different animals and their parts by communities. During the study, 36 key informants of either male or female were selected. From the included participants, most of them at least write and read, whereas 25% of them does not have no formal education. Most of the informants were aged individuals having age of 45 years (80.55%) and above. More than 80% of interviewed individuals are married and greater than 58% were living in the country side. Fifty five per cent of included traditional healers included in this survey were farmers with different educational backgrounds, whereas 19.4% of them were merchants (Table 1).

**TABLE 1:** Socio demographic factors of respondents.

Characteristics	Category	Frequency	Percentage
Sex	Male	21	58.33
	Female	15	41.67
Marital status	Married	29	80.55
	Unmarried	7	19.44
Residence	Rural	23	63.89
	Urban	13	36.11
Age in years	25–34	2	5.56
	35–44	5	13.89
	45–54	7	19.44
	55–64	12	33.33
	>65	10	27.78
Educational level	No formal education	9	25.00
	Read and write	12	33.33
	8–12 grade complete	7	19.44
	Diploma and above	8	22.22
Occupation	Farmer	20	55.56
	Merchant	7	19.44
	Human health professional	3	8.33
	Other professional	2	5.56
	Animal health professional	4	11.11
What are the main threats to use medicinal animals?	Deforestation	15	41.67
	Dependence on modern medicine	15	41.67
	Rules on animal welfare	6	16.67

### 3.2 Source of knowledge and its maintenance

From the study participants, the most common source of traditional knowledge was father (41.67%) followed by mother (25.0%). Regarding the reputability of the ethno medicinal practice 72.2% of the participants indicated that the knowledge is well accepted by the community, whereas 27.78% of informants showed the resistance of the community to use traditional medicinal animals. Relatively only few participants show interest to share their knowledge (38.89%) to other, whereas 61.11% of participants refuses to transfer their knowledge to others who seek to exercise this ethnozoological knowledge. From this study, it is learned that 47.22% of participants responded peoples can always use traditional medicine, whereas 36.11% of them reply as individuals use it situational. Economy and efficacy account above 61% of the reason why people use traditional medicine. Most of the healers (61.11%) responded as there is no conservation mechanism for the wealth of knowledge from traditional healers (Table 2).

**TABLE 2:** Source of knowledge, attitude and practice of the community related information (*n* = 36).

Variable		Frequency	Percentage
From where you acquire traditional healing?	Father	15	41.67
	Mother	9	25.00
	Grand parent	4	11.11
	Religious source	4	11.11
	Friends	2	5.56
	Trial and error	2	5.56
Is traditional healing practice accepted by the community	Accepted well	26	72.22
	Not accepted well	10	27.78
Does traditional healers interested to transfer medicinal knowledge to the next generation or others?	Interested	14	38.89
	Not interested	22	61.11
What benefit is gained from practicing traditional healing?	Income generation	6	16.67
	Satisfaction	7	19.44
	Self‐treatment (animal and family)	15	41.67
	Public service	8	22.22
Use of constant dose for similar disease	Yes	17	47.22
	No	19	52.78
How often people use traditional medicine	Some times	6	16.67
	Situational	13	36.11
	Always	17	47.22
What was the reason that forces the people to use traditional medicines?	Economy	11	30.56
	Lack of modern medicine	6	16.67
	Effectively	11	30.56
	Easy availably	8	22.22
Are there any conservation and documentation mechanism to this traditional medicinal knowledge?	Yes	8	22.22
	No	22	61.11
	To some extent	6	16.67

### 3.3 Type of medicinal animals

In the current study, from a total of 33 animal species identified as source of remedies for different human ailments, 63.63% were mammalian class, whereas 15.10% for birds. Insects, fish, reptiles and amphibians were told to be source of remedies. From these animals, 72.27% of them were wild, whereas 27.27% were domestic animals (Table 3).

**TABLE 3:** Class of animals used traditionally in the study area (*n* = 33).

SNO	Class of animals	Frequency	Percentage
1.	Mammals	21	63.63
2.	Birds	5	15.10
3.	Reptiles	3	9.09
4.	Amphibians	1	3.03
5.	Insects	3	9.09
6.	Fish	1	3.03
**Total**		33	100.00

### 3.4 Method of preparation and mode of application of medicinal animals

The medicinal animals were reported to be used in different routes of administration. The most common routes of administration were eating (43.0%) followed by drinking (28.0%), fumigation (12.00%) and heating (6.0%). Massaging, tying on, sitting on and hanging of medicinal products were reported to be the used routes of administration. The most common route of entry was found to be oral and dermal ways (Table 4).

**TABLE 4:** Mode of application (*n* = 100).

No	Mode of application	Number of application	Percentage	Route of entry
1.	Eating	43	43.00	Oral
2.	Drinking	28	28.00	Oral
3.	Tying	4	4.00	Dermal
4.	Anointing	4	4.00	Dermal
5.	Massaging	3	3.00	Dermal
6.	Fumigation	12	12.00	Nasal
7.	Heating	6	6.00	Dermal

This study revealed a total of 33 animal species under different genera and 6 classes which are used for treatments of 40 different human ailments. Wild mammals are the most commonly used classes followed by birds and insects, respectively. It delivered in different methods either fresh or being cooked, dried, smoked or mixed with other ingredients like sugar, butter, milk and salts (Table 5).

**TABLE 5:** Medicinal animals used to treat animal and human disease with mode of administration and method of preparation.

Local name in (Afan Oromo)	English common name	Scientific name	Habitat	Class	Indication	Parts used	Condition of preparation	Preparation type	Route of administration
Bararoo	Cockroach	*Periplaneta americana (linnaeus)*	Tame	Insect	Asthma	Whole body	Wings are removed, washed boiled and allowed to consume	Fresh	Oral
Nama	Human being	*Homo sapiens*	Tame	Mammal	Evil eye	Faeces	Drying the faeces of evil and smoking to affected person	Dry	Inhalation
					Evil eye	Saliva	Salivating on axe in favour of affected person	Fresh	‐
					Warts	Faeces	Applying fresh hot faeces on the edge and centre of warts	Fresh	Skin
Warabessa	Hyena	*Crocuta crocuta*	Wild	Mammal	Evil eye	Skin	Drying the skin and hanging in the home	Dry	‐
					Evil eye	Meat	Drying the leg meat and tying to neck	Dry	‐
					Evil spirit	Faeces	Mixing with water and ash then watering	Dry	Oral
					Stomach pain	Faeces	Mixing with water and ash then watering	Dry	Oral
					Evil sprit	Bone	Burning the bone and smoking to the affected person or house	Dry	Inhalation
Booyyee	Pig	*Sus scrofa domesticus*		Mammal	Headache and rheumatism	Meat	Consuming the fresh meat	Fresh	Oral
					Hepatitis	Meat, bile	Consuming uncooked meat or bile	Fresh	Oral
					Cancer	Liver	Eating fresh bile	Fresh	Oral
					Asthma	Bile	Consuming fresh bile	Fresh	Oral
					Cancer	Bile	Consuming fresh bile	Fresh	Oral
					Skin infection	Blood	Anointing the infected part	Fresh	Skin
					Arthritis	Meat	Eating the meat	Fresh	Oral
					Fattening of the animal	Meat	Feeding the meat to the animal	Fresh	Oral
					*Trypanosoma*	Blood	Allowing the animal to eat/drink the blood	Fresh	Oral
Xaddee	Porcupine	*Hystrix cristata*	Wild	Mammal	Tuberculosis	Meat	Eating the cooked meat for three days		Oral
					Asthma	Meat	Eating the cooked meat for 3 days		Oral
					Arthritis	Meat	Eating the meat	Fresh	Oral
					Chronic cough	Meat	Eating the meat for 3–5 days	Fresh	Oral
					Malaria	Meat	Feeding fresh meat	Fresh	Oral
					Kidney infection	Meat/bile	Eating meat or drinking bile	Fresh	Oral
					Hepatitis	Meat	Eating the meat for 3–5 days	Fresh	Oral
					HIV	Meat	Eating the fresh meat	Fresh	Oral
					Hepatitis	Liver	Eating fresh liver	Fresh	Oral
					Emaciation	Blood/meat	Eating the meat or drinking fresh blood	Fresh	Oral
Re'ee	Goat	*Capra hircus*	Tame	Mammal	Fighting dandruff	Faeces	Applying the dried powder over the head	Dry	Skin
					Snake bite	Milk	Drinking fresh milk	Fresh	Oral
					Febrile disease (mich)	Bile	Drinking the bile	Fresh	Oral
					Anaemia	Blood	Drinking the fresh blood	Fresh	Oral
Saawwa	Cow	*Bos indicus*	Tame	Mammal	Vision problem Malaria	Bile Bile	Massaging bile around closed eye Drinking the raw bile	Fresh Fresh	Intra ocular Oral
					Leg fracture	Leg	Leg prepared in soup form	Fresh	Oral
					Anaemia	Liver	Eating fresh liver	Fresh	Oral
					Gastritis, GIT problem	Yogurt	Eaten with fresh ‘enjera’ or bread or drunk	Fermented	Oral
					Snake venom	Milk	Milk is given immediately after snake bite	Fresh	Oral
					Wound	Urine	Adding and rinsing to the wound	Fresh	Skin/topical
					Abdominal pain	Milk	Drinking fresh milk	Fresh	Oral
Jaldessa	Monkey	*Papio anubis*	Wild	Mammal	Sleeping sickness	Faeces	Dry faecal matter is fumigated to the diseased person	Dry	Oral
					Febrile disease, eye disease, evil eye	Faeces	Mixing with water and drinking		Oral
					Fever	Urine	Drinking	Fresh	Oral
Qamalee	Grivet monkey	*Chlora*	Wild	Mammal	Chronic cough	Lung	Eating the fresh lung	Fresh	
					Bone fracture	Meat	Dressing the fatty meat around the fracture for a weak	Fresh	Topical
Kanniisa	Bee	*Apis mellifera*	Wild	Insects	Cancer	Whole animal	Whole honey bee is ground in water and prescribed to eat	‐	Oral
					Skin disease	Honey	Applying the honey on affected skin part		Topical
					Diarrhoea	Honey	A cup of hones is allowed to be eaten		Oral
					Hepatitis	Honey	Honey is allowed to be eaten		Oral
					Asthma	Honey	3–5 spoon full of honey is allowed to eat for a week every morning		Oral
					Chronic cough	Honey	Eating the hone morning	Fresh	Oral
Kannisa daamuu	Stingless bee	*Trigona* spp.	Wild	Insects	Asthma	Stingless bee honey	2–3 spoon full of stingless be honey is allowed to eat for a week every morning		Oral
					Appetite loss	Stingless bee honey	2–3 spoons of full of stingless be honey is allowed to eat every morning		Oral
Kuruphee	Bush duiker	Sylvica pragrimmia	Wild	Mammal	Arthritis, rheumatism	Meat	Eating cooked meat with ‘Enjera’ for 3 days	‐	Oral
					Hepatitis	Meat or blood	Eating the meat or drinking blood	Fresh	Oral
Bosonuu	Menelik's bushbuck	*Tragelaphus scriptus meneliki*	Wild	Mammal	Arthritis, rheumatism	Meat	Eating cooked meat with ‘Enjera’ for 3 days		Oral
					Sole infection	Fatty meat	Melting the fat on the affected area		Skin/topical
Jawwee	Python	*Phyton* spp.	Wild	Reptile	Tumour/leg swelling	Fatty meat	Applying/tying the fatty meat on the swelling for few days	‐	Skin/topical
					Alopecia	Fatty meat	Rubbing/massaging to the hair		Topical
					Bad sprit	Bone	Smoking the bone to the person affected		Inhalation
Bofa	Snake	*Snake* spp.	Wild	Reptile	Cancer/tumour	Whole animal	Applying the dead animal on tumour or the applying powder form on the swelling		Topical
					Wound	Whole	Drying and applying to the affected part	Dry	Topical
Lukkuu	Hen	*Gallusgallus*	Tame	Bird	Cough	Fat	The cooked hot fat is eaten		Oral
					Trough inflammation/nasal obstruction	Fat	The cooked hot fat is eaten		Oral
					Penile erection	Meat	Eating the well cooked meat for few days	Fresh	Oral
					Warts	Faeces	Fresh faeces is painted to the warts	Fresh	Skin
Raacha	Frog	*Rana* spp.	Wild	Amphibian	Asthma	Meat	Cooked meat is allowed to eat		Oral
Harree	Donkey	*Equus asinus*	Tame	Mammal	Ring worm	Milk	Drinking afresh milk for days	Fresh	Oral
					Alopecia	Blood	Bleeding the wart of donkey on the alopecia of man	Fresh	Topical
Wangoo	Fox	*Vulpes vulpes*	Wild	Mammal	Teeth	Epilepsy	Holding the teeth on epileptic patient	‐	Oral
Qotiyyoo	Ox	*Bos taurus*	Tame	Mammal	Liver	Anaemia	Eating the fresh liver	Fresh	Oral
					Skin	Swelling	Warming the swollen body by warmed skin	‐	Topical
					Bone/sole	Fracture	Eating the soup prepared from bone or internal part of the sole	‐	Oral
Qeerrensa	Tiger	*Panthera tigris*	Wild	Mammal	Rabies	Meat	Eating the dried meat for some days	Dry	Oral
Hoolaa	Sheep	*Ovis aries*	Tame	Mammal	Fat	Arthritis, swell, rheumatism	Massaging the affected area by fatty meat	Fresh	Topical
					Meat	Arthritis, swell, rheumatism	Eating the ‘wat’ prepared from meat for two consecutive days	Fresh	Oral
					Bile	Malaria	Drinking fresh bile	Fresh	Oral
					Blood	Anaemia	Drinking fresh blood	Fresh	Oral
					Milk	Malaria	Drinking fresh milk	Fresh	Oral
Simbirahalkanii	Bat	*Cynopterus sphinx*	Wild	Mammal	Whole	Wound around face	Rubbing/trying the dried bat body around the infection	Dry	Oral
Osolee	Squirrel	*Procavia capensis*	Wild	Mammal	Meat	Asthma	Boiled/cooked meat is allowed to eat	Fresh	Oral
					Meat	Heart disease	Eating the meat for affected person	Fresh	Oral
					Meat	Fattening	Providing the fresh meat to animals	Fresh	Oral
Karkarroo	Common warthog	*Phacochoerus africanus*	Wild	Mammal	Meat or liver	Inflammation/febrile disease	Eating cooked meat or fresh liver	Fresh	Oral
					Meat	Arthritis/rheumatism	Eating the meat	Fresh	Oral
					Teeth	Breast disease	Heating the breast with hot teeth	Dry	Topical
Naacha	Crocodile	*Crocodile* spp.	Wild	Reptile	Bile	Coughing, TB symptoms	Drinking the bile	Fresh	Oral
Wakkallee/illeettii	Rabbit	*Oryctolagus cuniculus*	Wild	Mammal	Skin	Menstruation disorder	Tying the skin	Dry	Topical
Leenca	Lion	*Panthera leo*	Wild	Mammal	Skin	Evil sprit	Smoking the dried skin	Dry	Topical
					Skin	Female sterility	Tying ‘kudhaamaa’ made of skin to the neck		
Roobii	Hippopotamus	*Hippopotamus amphibius*	Wild	Mammal	Bone	Beast swelling, sun burn and fracture	Tying to the swelling	Fresh	Topical
					Meat	Skin disease	Washing with soap and meat	Fresh	Topical
Simbirroo	Birds	*Alectoris rufa*	Wild	Bird	Egg	Cold disease	Drinking the egg	Fresh	Oral
Qurxummii	Fish		Wild	Fish	Meat	Asthma	Dry fish is grinded and prescribed to drink with water	Dry	Oral
Allaattii	Pigeon	*Columba arquatrix*	Wild	Bird	Blood	Burn	Applying the blood on the burned surface	Fresh	Topical
					Meat	Hepatitis	Eating the meat	Fresh	Topical
Gogorrii	Partridge	Pternistiserckelii	Wild	Bird	Meat	Asthma	Eating the cooked meat	Fresh	Topical

Preparation methods involve cooking, smoking, burning with, crushing, powdering, wrapping and the use of fresh animals or their products. It varies according to different ailments treated (Table 5). It is told in this study that meat and fat (33.33%) are the most frequently used animal part followed by liver and animal products such as honey, egg and milk butter. Whole animal, excrete; blood, teeth and blood are used for medicinal purposes. The preparation conditions various from using the animals or products fresh or drying and preparing in powder form (Table 6).

**TABLE 6:** Animal parts or products used for traditional medicine used for treatment of ailments (*n* = 102).

SNO	Medicinal parts or products of animals	No. of parts/products used	Percentage
1.	Meat/fat	34	33.33
2.	Visceral organ (liver)	11	10.78
3.	Bile	9	8.82
4.	Products (honey, egg, milk, butter, yoghurt)	16	15.69
5.	Bone/teeth	6	5.89
6.	External body parts (skin, external sole)	7	6.89
7.	Blood	7	6.89
8.	Whole animal body	3	2.94
9.	Excrete (urine and stool)	9	8.82

### 3.5 Relative importance and fidelity level of medicinal animals and their products

The present study revealed that *Hystrix cristata* with 36.11 use value percentage is the most frequently used animal species for different ailments followed by *Sus scrofa domesticus, Apis mellifera* and *Procavia capensis* (Table 7).

**TABLE 7:** Use value of medicinal animal for treatment of common disease in the study area.

SNO.	Local name in (Afan Oromo)	English common name	Scientific name	∑iUvi	Uv	% Uv
1.	Xaddee	Porcupine	*Hystrix cristata*	13	0.36	36.11
2.	Booyyee	Pig	*Sus scrofa domesticus*	10	0.27	27.80
3.	Kanniisa	Bee	*Apis mellifera*	9	0.25	25.00
4.	Osolee	Squirrel	*Procavia capensis*	9	0.22	22.22
5.	Kannisa daamuu	Stingless bee	*Trigona* spp.	8	0.22	22.22
6.	Hoolaa	Sheep	*Ovis aries*	6	0.17	16.70
7.	Saawwa	Cow	*Bos indicus*	5	0.13	13.89
8.	Simbirahalkanii	Bat	*Cynopterus sphinx*	5	0.13	13.89
9.	Roobii	Hippopotamus	*Hippopotamus amphibius*	5	0.13	13.89
10.	Lukkuu	Hen	*Gallus gallus*	4	0.11	11.11
11.	Wangoo	Fox	*Vulpes vulpes*	4	0.11	11.11
12.	Qurxummii	Fish	*Any fish species*	4	0.11	11.11
13.	Warabessa	Hyena	*Crocuta crocuta*	3	0.08	8.33
14.	Re'ee	Goat	*Capra hircus*	3	0.08	8.33
15.	Jawwee	Python	*Phyton* spp.	3	0.08	8.33
16.	Bofa	Snake	*Snake* spp.	3	0.08	8.33
17.	Qotiyyoo	Ox	*Bos Taurus*	3	0.08	8.33
18.	Naacha	Crocodile	*Crocodile* spp.	3	0.08	8.33
19.	Simbirroo	Birds	*Alectoris rufa*	3	0.08	8.33
20.	Kuruphee	Bush duiker	Sylvicapra grimmia	2	0.05	5.56
21.	Bosonuu	Menelik's bushbuck	*Tragelaphus scriptus meneliki*	2	0.05	5.56
22.	Harree	Donkey	*Equus asinus*	2	0.05	5.56
23.	Karkarroo	Common warthog	*Phacochoerus africanus*	2	0.05	5.56
24.	Wakkallee	Rabbit	*Oryctolagus cuniculus*	2	0.05	5.56
25.	Allaattii	Pigeon	*Columba arquatrix*	2	0.05	5.56
26.	Gogorrii	Partridge	*Pternisti serckelii*	2	0.05	5.56
27.	Bararoo	Cockroach	*Periplaneta americana (linnaeus)*	1	0.02	2.78
28.	Nama	Human being	*Homo sapiens*	1	0.02	2.78
19.	Jaldessa	Monkey	*Papio anubis*	1	0.02	2.78
30.	Qamalee	Grivet monkey	*Chlora*	1	0.02	2.78
31.	Raacha	Frog	*Rana* spp.	1	0.02	2.78
32.	Qeerrensa	Tiger	*Panthera tigris*	1	0.02	2.78
33.	Leenca	Lion	*Panthera leo*	1	0.02	2.78

*Note*: ∑iUvi: the sum of the number of use reports sited by the informants.

Abbreviation: Uv: use value, %Uv: use value percentage.

The FL of medicinal animal species was determined for the most commonly reported disease by the informants (Table 8). Accordingly, honey from bee and sting less bee species (*A. mellifera* and *Trigona* spp.) is known to relieve wart, asthma, diarrhoea, throat pain, stomachache, cough and tuberculosis and achieves 100% FL. In addition to this, *Sus scrofa domesticus*, *H. cristata*, urine of *Bos indicus* and *Crocuta crocuta* indicated 73%, 75%, 80% and 100% FL for the treatment of hepatitis, asthma, snake venom and evil spirit respectively (Table 8).

**TABLE 8:** Fidelity level of medicinal animals for treating common disease.

SNO.	English common name	Scientific name	Indication	No. of informants agreed for the indication	Total No. of informants using the animal	Fidelity level
1.	Porcupine	*Hystrix cristata*	Asthma	12	16	75.0
2.	Pig	*Sus scrofa domesticus*	Hepatitis	11	15	73.3
3.	Bee (honey)	*Apis mellifera*	Asthma	8	8	100
4.	Squirrel	*Procavia capensis*	Arthritis	7	9	77.8
5.	Stingless bee (honey)	*Trigona* spp.	Asthma	9	9	100.0
6.	Cow (milk)	*Bos indicus*	Snake venom	4	5	80.0
7.	Bat	*Cynopterus sphinx*	Viral skin infection	5	5	100.0
8.	Hen	*Gallus gallus*	Penile erection	2	4	50.0
9.	Common warthog	*Phacochoerus africanus*	Arthritis	2	5	20.0
10.	Hyena	*Crocuta crocuta*	Evil sprit	3	3	100.0
11.	Goat	*Capra hircus*	Snake bite	1	3	33.3
12.	Python	*Phyton* spp.	Tumour	2	3	66.7
13.	Snake	*Snake* spp.	Tumour	3	5	60.0
14.	Ox	*Bos taurus*	Anaemia	2	3	66.7

### 3.6 Information consensus factor

In this study, the level of agreement between interviewees over which animal to use for each illness category was determined using ICF. This study revealed that informants have high degree of agreement (ICF = 1) in the treatment of asthma, hepatitis and warts. However, the informants have a high level of heterogeneity (ICF = 0.5) in the treatment of evil eye and febrile disease (Table 9).

**TABLE 9:** Informant consensus factor for the common disease.

SNO	Disease treated	No. of use reports	No. of species for treated disease	Informant consensus factor
	Asthma	2	1	1
	Hepatitis	2	1	1
	Evil eye	2	1	0.5
	Warts	2	1	1
	Cancer	7	4	0.5
	Febrile disease	3	2	0.5
	Snake bite	3	2	0.5
	Emaciation	6	3	0.6
	Cough	4	2	0.7
	Anaemia	3	2	0.5
	Sterility	5	3	0.5

## 4 DISCUSSION

In the present ethnozoological survey, 33 animal species and their parts/products that belong to a class of mammals, birds, reptiles, amphibians and insects were reported to be used for the treatment of 40 kinds of human and different livestock health conditions by traditional medicinal practitioners of Jimma Arjo districts’ community. The current result is slightly lower to study report by Kendie et al. (2018) from Metema who indicated 51 medicinal animals. This finding is higher than study conducted in the semi‐arid regions of Northern Brazil that reported 25 medicinal animals species used for the treatment of 43 different ailments (Alves et al., 2012). Similarly, a study conducted in West Gojjam Ethiopia also revealed the use of 26 animal species for the treatment of 33 different ailments (Misganaw et al., 2021). Lower number of medicinal animals was reported from Arba Minch Zuria district (Kebebew et al., 2021) and Kafta‐Humera (Yirga et al., 2011) that reported the use of 19 and 16 animal species, respectively. In this study, majority of the medicinal animals (85%) were wild animals. This finding is in agreement with the study conducted in semi‐arid regions of Northern Brazil which reported wild animals as the major (77.7%) source of animal‐based complementary medicines (Alves et al., 2011). The finding of the study conducted by Kebebew et al. (2021) in Arba Minch district and Yirga et al. (2011) in Kafta‐Humera district of Northern Ethiopia reported more than half of medicinal animals were obtained from wild sources. This finding is in agreement to the reports by other researchers as wild and domestic animals and their products such as skin, bone, feather, tusk, hooves, blood, honey and others are important ingredients in the preparation of curative, protective and preventive medicine (Kindie et al., 2018)

This finding indicated that traditional medicinal practitioners and indigenous people are mostly dependent on the wild sources which might be related to the preference of the community for wild animals. Animals of all kinds are utilized for food, clothes and traditional health care practices to treat a variety of human and livestock illnesses, ranging from insect larvae to larger mammalian species.

In this study, mammals were the most commonly (63.6%) used class of animals followed by birds (15%) and reptiles, insects (9.09%). This finding is in line with the review conducted in the Mexican traditional system that reported mammals as the most commonly used medicinal class of animal species followed by birds and reptiles (Alonso‐Castro, 2014). The study conducted in the semi‐arid region of Northern Brazil also reported mammals as the most commonly used class of medicinal species (Alves et al., 2014). Similarly, the study conducted among the indigenous people of Metema Woreda, Northwestern Ethiopia also reported mammals as the most commonly used animal species followed by birds and reptiles (Kindie et al., 2018). Study conducted by Abebe et al. (2022) from Motta city administration and Hulet Eju Enessie and Kebebew et al. (2021) from Arba Minch Zuria district indicated similar value (64%) as mammals are most commonly used animal class (Abebe et al., 2022; Kebebew et al., 2021). In line with the current finding, the medicinal use of insects and other arthropods plays an important role to treat various maladies and injuries and has a long tradition can be effective and provide results (Meyer Rochow, 2017). Mantis egg is produced from preying female mantis, which have significant pharmacological effect and edible value (Chinese Pharmacopoeia Commission, 2015). It was used as diuretics to improve urinary dysfunction and exploited tonics for enhancing immunity (Jia et al., 2016). The *N*‐(3,4‐dihydroxyphenylethyl) acetamide and 2,4‐di‐*tert*‐butylphenol were used as strong antioxidants and proved potent candidate for against hyperlipidaemia and atherosclerosis (Xu, 2014). Cicada slough is often used in Chinese folk medicine to improve throat discomfort, relieve spasm, treating skin disease, immunomodulatory effect, combat allergies and esophagitis (Xu et al., 2006; Zhang, 2007). Honey bee and stingless bees are common traditional insects for treatment of arthritis, rheumatism, pain, tumour, skin disease and asthma. Rheumatoid arthritis is treated by bee venom as it induces apoptosis through caspase‐3 activation in synovial fibroblasts of patients (Hong et al., 2005). Maggot therapy is a type of biotherapy that involves in the introduction of live, disinfected maggots in to non‐healing skin and soft tissue for cleaning and debridement of dead necrotic tissue (Sherman & Pechter, 1988). The present study indicated that different parts/products of medicinal animals are used for their healing values. Accordingly meat or fatty meat was the most commonly (33.3%) followed by animal products such as milk, honey, egg and liver. Similarly, other studies also reported the meat/flesh of different animals as most commonly used for its medicinal value for the management of different ailments (Abebe et al., 2022; Erena, 2020; Kendie et al., 2018; Kebebew et al., 2021; Tesfaye & Erena, 2020; Yohannes & Chane, 2014). This study indicates the oral route either in the form of eating or drinking accounts (71.0%) for the administration of medicinal preparations followed by fumigation (12.0%). Similarly, other studies also reported the oral route as the major route for administration of the medicinal preparations (Abdeta et al., 2020; Abebe et al., 2022; Mahomoodally et al., 2019; Yirga et al., 2011). However, contrary to current study finding, the study conducted in Arba Minch Zuria district reported the dermal route as the major route compared to the oral route of administration (Kebebew et al., 2021).

The relative importance of a species cited by the informants was determined using use‐value. The present study reported that *H. cristata, Sus scrofa domesticus* and *A. mellifera* 36.11%, 27.8% and 25.0%, respectively, were most common cited medicinal animal. These higher values of some of species might be related to the preparation of different remedies from the different parts of a single animal species to treat different ailments (Alves et al., 2009). Most of the healer's individual's life is influence by beliefs about the magical power of the medicinal animals as in other part of the world.

The finding of this study indicated that most of the medicinal animal species are being lost due to deforestation and over‐exploitation. The loss of medicinal animals might be associated with slaughtering the animal species to collect the meat, organ, blood and other parts which were commonly reported to prepare most of the medicinal remedies. Today most of the community also uses ready available commercial drugs. In line to this, an attempt to conserve of these medicinal animals along their niches is rare from government and non‐government organization though the local wisdom of indigenous people in nature conservation plays a crucial part in protecting planets biodiversity and the overall health of the ecosystem.

### 4.1 Limitations of the study

The fact that the current study only gathers the essential data over a third of a year is one of its highlighted weaknesses. Furthermore, most of the traditional healers are not willing to show the exact mechanism how the medicine is prepared other than responding to the questions. This is from fear if they do that the knowledge will be transferred to anyone who seek seeks it, which will reduce their value in the community as every one become expert to the specific disease. Furthermore, it is very difficult to capture the every animal for further investigation as most the animals are not assessable. The prevalence of ailments and the accessibility of remedies both vary seasonally.

## 5 CONCLUSION

Developing countries commonly used traditional medicines as one of the alternative medicinal practices. In Jimma Arjo district, traditional medicinal practitioners and indigenous people practiced traditional medicine using animal based remedies. In this study, 33 animal species that belong to mammals, birds, reptiles, amphibians and insects were used for the management and treatment of different types of human and livestock ailments. Mammals were the most frequently used. Although the traditional medicinal practitioners and indigenous people are skilled with the preparation and administration of animal based remedies, less effort has been made to conserve the medicinal animals that lead the loss of knowledge and practice as the elders pass. Hence, it is important to document, conserve and manage; the indigenous knowledge and further research should be conducted to test the products scientifically for product development.

## AUTHOR CONTRIBUTIONS

Conceptualization; methodology; supervision; resource; supervision; project administration; analysis; writing the original draft and editing final paper.

## CONFLICT OF INTEREST STATEMENT

The author declares there is no conflict of interest

### PEER REVIEW

The peer review history for this article is available at https://www.webofscience.com/api/gateway/wos/peer‐review/10.1002/vms3.1277.

## ETHICS STATEMENT

Before starting data collection, ethical approval was obtained for oral informed consent from the research ethics committee of School of Veterinary Medicine, Wollega University dated 20/08/2020 with minute number SVM.RERC/0019., ethical approval was obtained for oral consent from the Research Ethics Committee of the School of Veterinary Medicine, Wollega University dated 20/08/2020 with minute number SVM.RERC/0019. Communicative letter was written to district and peasant association leaders explaining the objective of this study. Data was collected after the respondents agree to participate in the interview process. It is assured that information collected was anonymous and that it was only for research purpose. In this process, animals are not sacrificed rather the picture is taken at the spot.

## Data Availability

Data available on request from the authors.
